# Epigenetic Age Acceleration Is Associated With HIV Infection Independently of Inflammation

**DOI:** 10.1093/ofid/ofag270

**Published:** 2026-05-05

**Authors:** James K Gibb, Joshua M Schrock, Brian Mustanski, Thomas W McDade, Richard T D’Aquila

**Affiliations:** Department of Anthropology, Northwestern University, Evanston, Illinois, USA; Department of Anthropology, Indiana University, Bloomington, Indiana, USA; Kinsey Institute, Indiana University, Bloomington, Indiana, USA; The Irsay Institute for Sociomedical Sciences Research, Indiana University, Bloomington, Indiana, USA; Impact Institute, Northwestern University, Chicago, Illinois, USA; Division of Infectious Diseases, Department of Medicine, Feinberg School of Medicine, Northwestern University, Chicago, Illinois, USA; Impact Institute, Northwestern University, Chicago, Illinois, USA; Department of Medical Social Sciences, Northwestern University, Chicago, Illinois, USA; Department of Anthropology, Northwestern University, Evanston, Illinois, USA; Institute for Policy Research, Northwestern University, Evanston, Illinois, USA; Division of Infectious Diseases, Department of Medicine, Feinberg School of Medicine, Northwestern University, Chicago, Illinois, USA

**Keywords:** aging, epigenetics, HIV, inflammation, methylation

## Abstract

**Background:**

People with human immunodeficiency virus (HIV) (PWH) experience epigenetic age acceleration (EAA) and earlier onset of age-related comorbidities. Contributions to EAA from systemic inflammation versus HIV-specific effects warrant further study.

**Method:**

We analyzed peripheral-blood cell deoxyribonucleic acid (DNA) methylation (DNAm) in persons aged 18–30 years who were born with male sex. People with sexually acquired HIV who were on antiretroviral therapy (ART) with detectable plasma viral load, as is frequent in clinical practice in the United States, were matched on demographics, substance use, smoking, and inflammation to people without HIV who were at sexual risk of HIV. Systemic inflammation was stratified by plasma C-reactive protein (CRP; low/high). Epigenetic age estimates were regressed on chronological age to test associations with HIV status and inflammation.

**Results:**

PWH showed significantly greater EAA than people without HIV across multiple measures at both CRP levels (all *P* < .001). HIV status was strongly associated with higher EAA (eg, PC-Horvath1 β = 4.59 years; PC-Horvath2 β = 5.31; and PC-PhenoAge β = 5.54; all *P* < .001) and shorter DNAm-derived telomere length (β = −0.23; *P* < .001). Plasma CRP, interleukin-6, and lipopolysaccharide-binding protein did not explain these associations. Adjustment for methylation-derived naive and memory CD4/CD8 T-cell proportions substantially attenuated the association of EAA with HIV.

**Conclusions:**

Among young adult PWH with detectable viremia despite ART, EAA was more strongly associated with HIV infection than with systemic inflammation. Shifts in blood T-lymphocyte composition caused by HIV contributed to epigenetic aging observed here among PWH in a typical clinical setting in which optimal HIV suppression was not universally consistent.

## Graphical Abstract

**Graphical Abstract:**
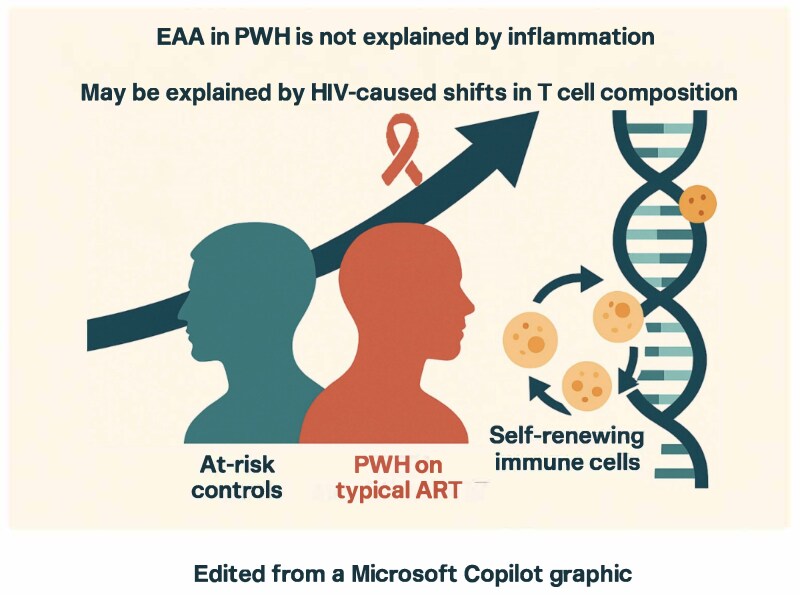
Epigenetic age was accelerated among young adults living with sexually acquired HIV on clinically typical suboptimal ART, relative to at-risk controls with similarly elevated systemic inflammation. Implications for research to counter premature biological aging in HIV are discussed, including evaluating a different setting with consistently optimal HIV suppression.

People with human immunodeficiency virus (HIV)-1 (PWH) experience earlier onset and increased frequency of aging-related adverse health outcomes than people without HIV, despite prevention of acquired immunodeficiency syndrome (AIDS) with antiretroviral therapy (ART) [1, 2]. These non–AIDS-defining comorbidities, thought to reflect premature biological aging, include hypertension, diabetes, cardiovascular disease, non–AIDS-defining cancers, cognitive decline, and reduced kidney, liver, lung, and bone functions [1, 2]. Epigenetic alterations are among several causal mechanisms of biological aging, referred to as “hallmarks of aging,” which are normally seen later in life among people without HIV. These also include cellular senescence and immune dysregulation [3–5]. In addition, systemic inflammation has been implicated in the pathogenesis of cardiovascular, neurocognitive, and other comorbidities later in life among people without HIV, is not fully normalized in PWH despite suppressive ART, and has been suggested to contribute to earlier comorbidities among ART-treated PWH [6].

Biological age is often measured as epigenetic age (EA) [7–9]. Epigenetic age has been assessed by quantifying the accumulation of methylation at CpG dinucleotides across the genome [9, 10]. Different “clocks” can identify epigenetic age acceleration (EAA) beyond chronological age and have been developed for different purposes or specific questions [10]. Epigenetic age is an inexorable consequence of normal organismal aging over time at either a similar or different pace in blood cells and/or different tissues of many PWH [7, 11]. Exposure to HIV as well as other biological, environmental, social, and behavioral factors can increase the pace at which the epigenetic clock “ticks”; prior reviews detail these effects and different analytic uses of several clocks [12–14]. Epigenetic age acceleration begins at earlier chronological ages among PWH than among people without HIV, occurring by 3 years, if not sooner, after HIV acquisition in the absence of ART [15].

The “ticking” may slow during viremia-suppressing ART, albeit with heterogeneity both across individuals and over time within an ART-treated individual [15–18]. Prior studies that examined EA in PWH cohorts with near-universal viral suppression on ART (83%–93% undetectable) also reported EAA [19, 20]. Persisting EAA during suppressive ART could be consistent with a larger mechanistic role for the systemic inflammation that persists during ART than for any effects of ongoing HIV replication that ART suppresses. Cumulative social disadvantages have been linked to elevated systemic inflammation [21, 22]. Substance use, trauma, stigma and discrimination, dysbiosis, and diet can each enhance inflammation, and people with behaviors associated with HIV acquisition risk are often exposed to these factors, even if not infected. Indeed, we previously reported similarly elevated levels of systemic inflammation among both PWH and at-risk people without HIV in the cohort of 18- to 30-year-olds studied here [23, 24].

Studying participants in this cohort also allowed us to evaluate EAA in a “real-world” ART context. In clinical practice in the United States, nonadherence to ART has been documented on 20%–60% of prescribed days, despite self-reported adherence [25–27]. Such intermittent adherence can lead to at least temporarily detectable viral load. The US-based cohort of young adults with HIV studied here demonstrates this, as detectable viremia was documented on 46% of visits among those on ART, with 73% concordance between self-report and laboratory documentation of undetectability [28]. Thus, we compared PWH with detectable viremia despite ART with people without HIV who had similar HIV acquisition risk behaviors and elevated systemic inflammation at the cohort entry visit. We found that HIV status was associated with EAA, whereas systemic inflammation was not.

## METHODS

### Study Population

Participants were enrolled in RADAR, an observational cohort study of people at risk of acquiring HIV or with sexually acquired HIV [23, 24, 28, 29]. All participants in RADAR were aged 18–30 years and were assigned male sex at birth; participants with HIV acquired HIV during adolescence or young adulthood. For this specific study, blood cells and plasma collected at the cohort entry visit were analyzed from a subsample of 50 PWH and 50 people without HIV to examine associations among EAA, HIV status, and systemic inflammation. Levels of the plasma inflammation biomarker C-reactive protein (CRP) were used to categorize systemic inflammation. People with HIV were selected based on self-reported ART use and detectable plasma viremia at the entry visit. Propensity scoring was used to match people without HIV to PWH on the following variables: inflammation, age, race/ethnicity, substance use, marijuana use, alcohol use, and smoking status. The subsample included 25 PWH with low inflammation (untransformed CRP < 1.54 mg/L), 25 PWH with high inflammation (untransformed CRP > 3.16 mg/L), 25 people without HIV with low inflammation (untransformed plasma CRP < 1.54 mg/L), and 25 people without HIV with high inflammation (untransformed CRP > 3.16 mg/L). Each participant with HIV self-reported time since HIV diagnosis. CD4 counts and viral load were determined. All study protocols were approved by the Institutional Review Board at Northwestern University.

### HIV Testing

Fingerstick blood samples were tested using the Alere Determine HIV1/2 Ab/Ag Combo fourth-generation point-of-care test from each participant previously seronegative for HIV at visits occurring every 6 months. Those who tested positive on point-of-care HIV testing received confirmatory HIV antigen and antibody immunoassay testing following current Centers for Disease Control and Prevention (CDC) HIV testing guidelines [30].

### Plasma Biomarkers of Inflammation and Microbial Translocation

Venous blood samples were assayed for 2 plasma biomarkers of inflammation: CRP (dynamic range, 0.001–49.6 mg/L, high-sensitivity assay) and interleukin-6 (IL-6; dynamic range, 0.06–488 pg/mL). Each was quantified via electrochemiluminescence using the MESO QuickPlex SQ 120 system following the manufacturer's protocol (Meso Scale Diagnostics, Rockville, MD). Plasma lipopolysaccharide-binding protein (LBP; dynamic range, 101-X pg/mL), which reflects circulating bacteria-derived lipopolysaccharide, was measured using the MSD R-PLEX Human LBP assay.

### DNA Methylation and Measures of Epigenetic Aging

Deoxyribonucleic acid (DNA) methylation (DNAm) was assessed using the Infinium Human MethylationEPIC v2.0 BeadChip array (Illumina, Inc., CA, USA) in the NUSeq Core Facility at Northwestern University Feinberg School of Medicine. A 500-ng DNA sample, extracted and cryopreserved from whole-blood cells (QIASymphony SP, DNA Midi kit, Qiagen, Inc.) at the study entry visit, was used to perform bisulfite conversion followed by Illumina's protocol for methylation profiling. BeadChips were scanned with an Illumina iScan and then analyzed using Illumina GenomeStudio software. BeadChip data were checked for quality by calculating detection *P*-values using the minfi package in R [31]. The NOOB normalization method via MePylome was then performed on the data to account for sample-to-sample variation. Low-quality probes were filtered from the dataset, along with probes with single nucleotide polymorphisms at the CpG site, probes that bind to multiple locations, and probes on sex chromosomes.

The calcPCClocks function from the PC-Clocks package in R was used to calculate EA using the following principal component-based clocks: PC-Horvath1, PC-Horvath2, PC-Hannum, and PC-PhenoAge [32]. We also calculated PC-DNAm telomere length, which is a methylation-derived proxy for telomere length rather than a measure of EA; shorter calculated telomere length is associated with a more rapid pace of aging. For each epigenetic clock-based measure, residuals from regression models of the biological age measure on chronological age (age in years at the time of blood sampling) were used to calculate EAA [10]. In addition, we calculated DunedinPACE, a DNAm-based measure of the pace of biological aging rather than an estimate of biological age in years. DunedinPACE is scaled such that values around 1.0 reflect the reference cohort's average pace of aging, and values >1.0 indicate faster aging (ie, a higher rate of physiological decline) [33]. Because DunedinPACE is a rate metric rather than an age estimate, we modeled DunedinPACE directly rather than deriving age-acceleration residuals from regression on chronological age.

### Data Analysis

Descriptive statistics, including mean and standard deviation, were computed for all continuous variables. C-reactive protein, IL-6, and LBP levels were natural log transformed prior to analysis as continuous variables. Frequencies and percentages were generated for categorical variables. We used Kruskal–Wallis tests to compare outcomes across the 4 CRP/HIV groups; to account for multiple testing across epigenetic measures, *P*-values were Bonferroni adjusted.

In addition to stratified group comparisons, we fit pooled regression models across all participants to (1) estimate HIV-associated differences with greater precision and (2) evaluate ln-transformed CRP as a continuous predictor rather than using dichotomized strata. Primary models included HIV status and ln-transformed high sensitivity (hs)CRP as predictors, consistent with the inflammation-stratified sampling design. Sensitivity models additionally adjusted for sociodemographic and behavioral covariates (chronological age, race/ethnicity, smoking status, marijuana use, substance use, alcohol use, depressive symptoms, perceived stress, and body mass index).

To evaluate whether blood immune cell composition explained associations between HIV status and epigenetic aging, proportions of leukocyte subsets (naive and memory CD4 T lymphocytes, naive and memory CD8 T lymphocytes, regulatory T cells, naive and memory B lymphocytes, and natural killer cells) were derived bioinformatically from DNAm data using a cell deconvolution algorithm [34]. Because estimated cell proportions are correlated and sum to 1.0, we summarized cell composition using principal components analysis (PCA) and included the first 4 principal components in regression models (selected a priori based on variance explained and interpretability). Models were also fit that adjusted only for naive and memory CD4+ and CD8+ T-cell proportions, given documented HIV-related alterations in these cell populations. When modeling cell-type proportions, we assessed multicollinearity using variance inflation factors and excluded terms with elevated collinearity to avoid overfitting.

Forest plots were generated using the sjPlot package [35]. For comparability of effect sizes across outcomes, standardized coefficients were computed by rescaling predictors by 2 SDs. Statistical significance was set at *P* < .05, and all analyses were conducted in R.

### Patient Consent Statement

All participants provided informed written consent. All study procedures were approved by Northwestern University's Institutional Review Board under protocol STU00087614.

## RESULTS

### Participants

People with HIV were well matched to people without HIV (Table 1). No differences were observed by HIV status and low versus high CRP levels in age, viral load, smoking, substance use, AUDIT score, or depression score (Supplementary Table 1). Among PWH, the median plasma viral load, reported ART use, and duration of infection did not differ between the 2 CRP strata (Supplementary Table 1). There was some variation across the 4 groups defined by HIV status and CRP level in race/ethnicity, plasma biomarker levels, self-reported marijuana use, and body mass index (Supplementary Table 1).

**Table 1.** Descriptive Statistics of Study Sample Stratified by HIV Status

Characteristic	Overall N = 100^a^	People Without HIV N = 50^a^	PWH N = 50^a^	*P-*Value^b^
Age	24.16 (2.84) 24.34 [18.05, 29.64]	23.87 (3.07) 23.80 [18.05, 29.64]	24.45 (2.60) 24.45 [18.54, 29.10]	.31
Race/ethnicity				.36
Non-Hispanic Black	75 (75.00%)	35 (70.00%)	40 (80.00%)	
Hispanic/Latinx	25 (25.00%)	15 (30.00%)	10 (20.00%)	
Substance use				>.99
No	63 (63.00%)	32 (64.00%)	31 (62.00%)	
Yes	37 (37.00%)	18 (36.00%)	19 (38.00%)	
Marijuana use				>.99
No	20 (20.00%)	10 (20.00%)	10 (20.00%)	
Yes	80 (80.00%)	40 (80.00%)	40 (80.00%)	
Current smoker				>.99
No	57 (57.00%)	29 (58.00%)	28 (56.00%)	
Yes	43 (43.00%)	21 (42.00%)	22 (44.00%)	
Ln-transformed high-sensitivity C-reactive protein	0.72 (1.82) 0.79 [−6.24, 4.55]	0.75 (1.79) 0.78 [−3.00, 4.55]	0.69 (1.86) 0.79 [−6.24, 3.63]	.92

^a^Mean (SD) median [min, max]; n (%).

^b^Kruskal–Wallis rank sum test; Pearson's chi-squared test.

### HIV Status and Not Plasma C-Reactive Protein Level Was Associated With Epigenetic Age Acceleration

HIV status was associated with EAA at each level of plasma CRP using multiple clocks (Kruskal–Wallis testing with Bonferroni correction, comparing PWH with the reference group of people without HIV at each dichotomized plasma CRP level; Table 2). High plasma CRP was not associated with EAA among people without HIV (Table 2).

**Table 2.** PWH Exhibit Epigenetic Age Acceleration (EAA) Relative to PWOH at Each Plasma CRP Level

Characteristic	Overall N = 100^a^	People Without HIV, Low CRP N = 25^a^	People Without HIV, High CRP N = 25^a^	PWH, Low CRP N = 25^a^	PWH, High CRP N = 25^a^	*P-*Value^b^
PC-based Horvath1 EAA	0.00 (4.67) 0.09 [−10.58, 9.97]	−2.56 (3.79)−2.75 [−10.45, 5.39]	−2.01 (4.08) −1.48 [−10.58, 3.94]	2.19 (4.79) 1.37 [−9.79, 8.73]	2.37 (3.78) 1.98 [−4.71, 9.97]	<.001
PC-based Horvath2 EAA	0.00 (5.28) 0.14 [−12.38, 11.86]	−3.02 (4.12) −3.03 [−11.45, 5.63]	−2.27 (4.92) −1.58 [−12.38, 5.12]	2.35 (5.11) 3.00 [−9.94, 10.78]	2.94 (4.26) 2.30 [−4.50, 11.86]	<.001
PC-based Hannum EAA	0.00 (4.57) −0.26 [−16.64, 9.96]	−1.86 (3.16) −1.20 [−7.67, 4.21]	−1.61 (4.36) −1.44 [−12.31, 5.29]	1.51 (5.51) 1.56 [−16.64, 9.06]	1.96 (3.78) 1.65 [−3.61, 9.96]	<.001
PC-based PhenoAge EAA	0.00 (5.93) 0.27 [−20.35, 11.43]	−3.31 (3.86) −2.21 [−9.56, 6.42]	−2.19 (5.74) −0.29 [−17.67, 5.87]	1.95 (6.50) 2.49 [−20.35, 10.97]	3.55 (4.61) 3.71 [−4.85, 11.43]	<.001
DunedinPACE (years)	1.03 (0.11) 1.04 [0.78, 1.28]	0.97 (0.12) 0.98 [0.78, 1.28]	1.05 (0.11) 1.09 [0.78, 1.21]	1.06 (0.10) 1.04 [0.88, 1.24]	1.05 (0.07) 1.05 [0.95, 1.20]	.009
PC-based DNAm telomere length	0.00 (0.19) 0.02 [−0.52, 0.37]	0.12 (0.11) 0.13 [−0.08, 0.36]	0.11 (0.10) 0.12 [−0.03, 0.37]	−0.09 (0.19) −0.10 [−0.42, 0.29]	−0.14 (0.17) −0.13 [−0.52, 0.17]	<.001

^a^Mean (SD) median [min, max].

^b^Kruskal–Wallis rank sum test, with Bonferroni correction.

**Figure 1.**
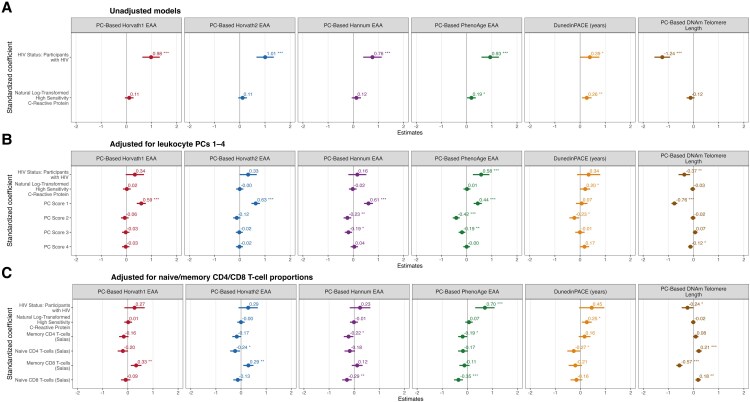
Regression models of epigenetic age acceleration (EAA). Models use six different metrics: A. without adjustment, B. with adjustment for leukocyte principal components (PCs) 1 through 4, and C. with adjustment for naive/memory CD4/CD8 T cell proportions.

All PWH were also compared with all people without HIV across both plasma CRP strata. HIV status was associated with EAA in these regression analyses using each clock (Table 3; Figure 1*A*). Continuous plasma CRP values were not associated with EAA in regression models including all PWH and people without HIV (Table 3; Figure 1*A*).

**Table 3.** Unadjusted Regression Models Show EAA Among All PWH Relative to All People Without HIV

	*Dependent Variable:*					
	PC-Horvath 1 EAA	PC-Horvath 2 EAA	PC-Hannum EAA	PC-PhenoAge EAA	DunedinPACE (years)	PC-DNAm Telomere Length
	(1)	(2)	(3)	(4)	(5)	(6)
HIV status: participant with HIV (ref. Participant without HIV)	4.585***	5.313***	3.490***	5.535***	0.041*	−0.230***
	(0.816)	(0.915)	(0.847)	(1.035)	(0.020)	(0.029)
Ln-transformed high-sensitivity CRP	0.287	0.318	0.312	0.616*	0.015**	−0.012
	(0.225)	(0.253)	(0.234)	(0.286)	(0.006)	(0.008)
Constant	−2.499***	−2.886***	−1.970**	−3.211***	1.003***	0.124***
	(0.602)	(0.674)	(0.624)	(0.763)	(0.015)	(0.021)
Observations	100	100	100	100	100	100
Log likelihood	−281.996	−293.404	−285.680	−305.737	87.618	51.571
Akaike inf. crit.	569.991	592.809	577.360	617.473	−169.237	−97.142

*P < .05; **P < .01; ***P < .001.

We next sought to explain the association of HIV status with EAA. Adjustment of models comparing all PWH with all people without HIV across both CRP strata by CRP, IL-6, or LBP, individually or together, did not alter the association of EAA with HIV status (results for IL-6 and LBP not shown). Adjusting for sociodemographic, behavioral, and body mass index covariates also did not materially alter the association of EAA with HIV status shown in Table 3 (Supplementary Table 2). These adjusted models included a binary indicator of substance use. Substance use did not change either the association of EAA with HIV status or the lack of association with inflammation, although it was associated with modestly lower EAA for several clocks (Supplementary Table 2).

### Blood Cell-Type Composition Explained the Association of HIV Status With EAA

A substantial contribution of blood cell composition to the variance in EAA was identified in PCA, consistent with prior evidence that HIV pathogenesis decreases immune cell self-renewal (Supplementary Figure 1) [36]. The first principal component (PC1) was itself associated with EAA (Figure 1*B*; Supplementary Table 3), and adjustment for the 4 predominant principal components substantially attenuated the association of HIV status with EAA (Figure 1*B* compared with Figure 1*A*; Supplementary Table 3 compared with Table 3). Adjusting for sociodemographic, behavioral, and body mass index covariates, including substance use, did not materially alter the effects of the 4 principal components (Supplementary Tables 3 and 4). Inspection of PC loadings indicated that PC1 primarily captured variation in T-lymphocyte composition relevant to HIV immunopathogenesis, with strong contributions from naive and memory CD4+ and CD8+ T-cell proportions (Supplementary Figure 1). Higher PC1 scores corresponded to redistribution across these naive and memory compartments, consistent with known HIV-associated remodeling of these immune cell populations. Because these cell subsets also correlated with EA measures, we evaluated whether adjustment for naive and memory CD4/CD8 proportions attenuated HIV-associated EAA. Only 1 of the 5 clocks (PhenoAge) retained an association, although attenuated, between EAA and HIV status after adjusting for the 4 lymphocyte subtypes (Figure 1*C* compared with Figure 1*A*; Supplementary Table 5 compared with Table 3). These findings indicate that differences in naive and memory lymphocyte proportions contributed to EAA among PWH.

## DISCUSSION

HIV status, and not systemic inflammation, was associated with EAA in this comparison of 18- to 30-year-old PWH selected for detectable viremia while receiving ART and matched people without HIV at 1 time point. Both groups shared HIV acquisition risk-related behaviors and had a similar range of plasma biomarker levels. People with HIV had pronounced EAA regardless of inflammation level. Epigenetic age acceleration was observed among PWH without high plasma CRP. In regression models including all PWH and people without HIV, HIV was associated with EAA. This pattern was consistent across multiple principal component-based epigenetic clocks, a principal component-based DNAm-derived telomere length measure, and DunedinPACE, which measures the pace of aging rather than biological age in years. The concordance across these measures supports an association between HIV and EAA.

In contrast, people without HIV with high plasma CRP did not exhibit EAA, nor was CRP as a continuous variable associated with EAA in analyses using any of the principal component-based clocks or DunedinPACE across all PWH and people without HIV. In addition, the association of EAA with HIV status was not explained by 2 alternate biomarkers reflecting, respectively, inflammation or microbial translocation, plasma IL-6 and LBP. Neither the association with HIV nor the lack of association with CRP was explained by sociodemographic and behavioral covariates, including substance use.

Our findings align with prior work showing EAA among PWH compared with HIV-negative controls, including studies using at-risk controls and studies using general-population controls without similar HIV acquisition risk [15–20, 37]. The results extend this literature to young adults with sexually acquired HIV and, importantly, evaluate systemic inflammation as a potential explanation for HIV-associated EAA in a cohort where PWH and people without HIV shared risk-related exposures. We did not observe evidence that inflammation, as operationalized by ln-transformed CRP, explained HIV-associated EAA in this young adult sample, although systemic inflammation has been clearly implicated in age-related comorbidities among older PWH. It is possible that an association of inflammation with EAA may be seen among those with longer duration of infection and/or older age.

Future research can address other alternative explanations for these findings and some limitations of the current work. Some community-based studies document substantial variation in associations of inflammation with disease, indicating the importance of longitudinal study of prolonged, uninterrupted inflammation rather than 1 time point, as well as the study of the complex interactions of inflammation with nutritional, microbial, and psychosocial environments [38]. Further, CRP may not capture the inflammation-triggered pathways most relevant to DNAm aging metrics in this context, which may include processes, such as coagulation, monocyte activation, or mitochondrial dysfunction. It is also conceivable that between-group differences in systemic inflammation among these PWH and people without HIV who shared HIV acquisition risk behaviors were insufficient to account for the observed differences in epigenetic aging. Larger and longitudinal studies with broader profiling of inflammation among populations with larger differences in inflammation and/or different environmental exposures could address these limitations. Given reported links between inflammation and immune cell senescence, future studies of cellular markers of immunosenescence, such as KLRG1 and CD57, are also of interest [39]. Future evaluation of NAD + regulation in HIV-associated EAA is also warranted, since T-cell CD38 ectoenzyme-mediated NAD+ decline can be induced by inflammatory mediators and reversed by an inhibitor of CD38 [40–43].

Of note, the association of EAA with HIV status was substantially decreased by adjusting either for all major variance-explaining principal components or only for the proportions of naive and memory subtypes of CD4 and CD8 T cells represented in PC1 (Figure 1*B* and *C*). We suggest that these findings, based on bioinformatic estimation of immune cell proportions from whole-blood methylation data, be confirmed in future studies using longitudinal DNAm profiling of isolated, immunophenotyped cell subpopulations. Such work can also evaluate whether partial restoration of naive T-cell pools on ART could slow EAA, even when CRP does not change.

Pharmacologic control of systemic inflammation among PWH receiving ART has had limited success to date in forestalling accelerated aging [2, 26]. This could be consistent with the greater contribution of mechanisms unique to HIV infection itself among PWH receiving ART, such as immune cell dysregulation and impaired T-cell self-renewal. Continued discovery and development of approaches to preventing HIV-related loss of immune adaptability and reparative capacity, along with continued efforts to address inflammation, are suggested by this work. Given that the ART-treated PWH studied here had detectable plasma viral load at study entry (Supplementary Table 1), it would be informative in future work to compare this group with PWH who achieve sustained viral suppression. This could include a study similar to the current one in a healthcare system outside the US that provides universal, consistent ART access and viral suppression. Such a study would enable evaluation of whether consistently optimal HIV suppression improves T-cell self-renewal and ameliorates EAA. If so, improving “real-world” clinical practice in the United States to increase early and consistently optimal viral suppression across affected populations could be studied as a way to better prevent HIV-associated EAA. Another strategy to counter EAA could be to replenish hematopoietic stem and progenitor cell renewal capacity. Approaches to eliminate antigen-expressing, immune cell-activating HIV reservoirs may also contribute to repairing deficits in immune cell self-renewal that lead to premature biological aging while also advancing the goal of curing HIV.

## Supplementary Material

ofag270_Supplementary_Data

## Supplementary Data

Supplementary materials are available at *Open Forum Infectious Diseases* online. Consisting of data provided by the authors to benefit the reader, the posted materials are not copyedited and are the sole responsibility of the authors, so questions or comments should be addressed to the corresponding author.
