# Supplementary material for: Epigenetic Age Acceleration Is Associated With HIV Infection Independently of Inflammation
Source: Open Forum Infect Dis. 2026 May 5;13(5):ofag270. doi: 10.1093/ofid/ofag270 (PMC13195299; doi:10.1093/ofid/ofag270)
Supplement: ofag270_Supplementary_Data [file ofag270_supplementary_data.zip › RADAR EEA Supplementary Materials_revised[72].docx]

**Supplementary Materials**

**Supplementary Table 1. Descriptive Statistics for RADAR Study Sample Participants (N=100)**

| **Characteristic** | **Overall** N = 100*^1^* | **PWOH, Low CRP** N = 25*^1^* | **PWOH, High CRP** N = 25*^1^* | **PWH, Low CRP** N = 25*^1^* | **PWH, High CRP** N = 25*^1^* | **p-value***^2^* |
| --- | --- | --- | --- | --- | --- | --- |
| **Age** | 24.16 (2.84) 24.34 [18.05, 29.64] | 23.42 (3.17) 22.96 [18.23, 29.64] | 24.31 (2.96) 24.04 [18.05, 28.52] | 24.90 (2.72) 24.66 [18.71, 28.73] | 24.00 (2.44) 24.01 [18.54, 29.10] | 0.27 |
| **Race/Ethnicity** |  |  |  |  |  | 0.049 |
| Non-Hispanic Black | 75 (75.00%) | 20 (80.00%) | 15 (60.00%) | 23 (92.00%) | 17 (68.00%) |  |
| Hispanic/Latinx | 25 (25.00%) | 5 (20.00%) | 10 (40.00%) | 2 (8.00%) | 8 (32.00%) |  |
| **Ln-Transformed High Sensitivity C-Reactive Protein** | 0.72 (1.82) 0.79 [-6.24, 4.55] | -0.79 (0.98) -0.63 [-3.00, 0.37] | 2.29 (0.80) 2.19 [1.19, 4.55] | -0.78 (1.47) -0.18 [-6.24, 0.43] | 2.16 (0.67) 2.18 [1.15, 3.63] | <0.001 |
| **Ln-Transformed Interleukin 6** | -0.33 (0.82) -0.37 [-2.41, 2.38] | -1.07 (0.57) -1.13 [-2.41, -0.08] | 0.10 (0.87) -0.18 [-0.94, 2.38] | -0.43 (0.65) -0.61 [-1.76, 1.51] | 0.08 (0.57) 0.04 [-1.28, 1.70] | <0.001 |
| **Ln-Transformed Lipopolysaccharide-binding protein** | 1.58 (1.76) 1.86 [-9.21, 3.27] | 1.37 (1.28) 1.80 [-3.19, 2.25] | 1.93 (2.35) 2.38 [-9.21, 3.27] | 1.58 (0.39) 1.65 [0.77, 2.42] | 1.44 (2.30) 1.87 [-9.21, 2.86] | <0.001 |
| **Ln-Transformed Viral Load** | 4.29 (0.71) 4.21 [3.25, 5.86] | NA (NA) NA [Inf, -Inf] | NA (NA) NA [Inf, -Inf] | 4.20 (0.78) 4.07 [3.32, 5.86] | 4.37 (0.63) 4.37 [3.25, 5.76] | 0.26 |
| Missing | 50 | 25 | 25 | 0 | 0 |  |
| **HV Status** |  |  |  |  |  | <0.001 |
| PWOH | 50 (50.00%) | 25 (100.00%) | 25 (100.00%) | 0 (0.00%) | 0 (0.00%) |  |
| PWH | 50 (50.00%) | 0 (0.00%) | 0 (0.00%) | 25 (100.00%) | 25 (100.00%) |  |
| **Current Smoker** |  |  |  |  |  | 0.29 |
| No | 57 (57.00%) | 13 (52.00%) | 16 (64.00%) | 11 (44.00%) | 17 (68.00%) |  |
| Yes | 43 (43.00%) | 12 (48.00%) | 9 (36.00%) | 14 (56.00%) | 8 (32.00%) |  |
| **Marijuana use** |  |  |  |  |  | 0.037 |
| No | 20 (20.00%) | 1 (4.00%) | 9 (36.00%) | 4 (16.00%) | 6 (24.00%) |  |
| Yes | 80 (80.00%) | 24 (96.00%) | 16 (64.00%) | 21 (84.00%) | 19 (76.00%) |  |
| **Substance use** |  |  |  |  |  | 0.47 |
| No | 63 (63.00%) | 15 (60.00%) | 17 (68.00%) | 13 (52.00%) | 18 (72.00%) |  |
| Yes | 37 (37.00%) | 10 (40.00%) | 8 (32.00%) | 12 (48.00%) | 7 (28.00%) |  |
| **AUDIT Score** | 5.32 (5.97) 3.00 [0.00, 35.00] | 4.68 (5.99) 3.00 [0.00, 23.00] | 4.36 (4.02) 3.00 [0.00, 15.00] | 6.24 (5.79) 4.00 [0.00, 23.00] | 6.00 (7.65) 4.00 [0.00, 35.00] | 0.53 |
| **Viral load status** |  |  |  |  |  | <0.001 |
| Undetectable | 50 (50.00%) | 25 (100.00%) | 25 (100.00%) | 0 (0.00%) | 0 (0.00%) |  |
| Detectable | 50 (50.00%) | 0 (0.00%) | 0 (0.00%) | 25 (100.00%) | 25 (100.00%) |  |
| **Viral load (copies/mL)** | 71,015.72 (139,963.30) 16,240.00 [1,771.00, 726,651.00] | NA (NA) NA [Inf, -Inf] | NA (NA) NA [Inf, -Inf] | 78,997.52 (160,968.66) 11,828.00 [2,072.00, 726,651.00] | 63,033.92 (118,118.82) 23,593.00 [1,771.00, 574,916.00] | 0.26 |
| Missing | 50 | 25 | 25 | 0 | 0 |  |
| **CD3/CD4 percent** | 22.94 (9.64) 22.00 [9.10, 46.54] | NA (NA) NA [Inf, -Inf] | NA (NA) NA [Inf, -Inf] | 23.51 (10.09) 22.10 [12.70, 46.54] | 22.37 (9.35) 21.90 [9.10, 45.40] | 0.75 |
| Missing | 50 | 25 | 25 | 0 | 0 |  |
| **CD3/CD4 absolute count** | 416.04 (215.87) 352.50 [62.00, 1,060.00] | NA (NA) NA [Inf, -Inf] | NA (NA) NA [Inf, -Inf] | 378.76 (143.89) 361.00 [142.00, 664.00] | 453.32 (267.47) 344.00 [62.00, 1,060.00] | 0.57 |
| Missing | 50 | 25 | 25 | 0 | 0 |  |
| **Are you currently taking any HIV medication?** |  |  |  |  |  | >0.99 |
| Yes | 18 (46.15%) | 0 (NA%) | 0 (NA%) | 9 (45.00%) | 9 (47.37%) |  |
| No | 21 (53.85%) | 0 (NA%) | 0 (NA%) | 11 (55.00%) | 10 (52.63%) |  |
| Missing | 61 | 25 | 25 | 5 | 6 |  |
| **Estimated years since HIV diagnosis** | 3.36 (3.07) 2.70 [0.00, 12.50] | NA (NA) NA [Inf, -Inf] | NA (NA) NA [Inf, -Inf] | 4.15 (3.45) 4.20 [0.00, 12.50] | 2.58 (2.47) 2.10 [0.00, 9.90] | 0.091 |
| Missing | 50 | 25 | 25 | 0 | 0 |  |
| **PROMIS Depression T Score** | 51.63 (10.87) 53.20 [38.20, 75.00] | 54.26 (11.58) 57.70 [38.20, 75.00] | 51.32 (9.76) 52.10 [38.20, 68.70] | 51.78 (11.20) 53.20 [38.20, 71.70] | 49.14 (10.91) 52.10 [38.20, 73.90] | 0.40 |
| **PSS Sum Score** | 17.59 (6.87) 19.00 [0.00, 34.00] | 19.36 (6.63) 20.00 [5.00, 34.00] | 17.44 (7.26) 18.00 [0.00, 30.00] | 18.44 (6.49) 19.00 [3.00, 30.00] | 15.12 (6.75) 17.00 [1.00, 24.00] | 0.20 |
| **BMI** | 25.85 (6.25) 23.86 [16.78, 49.42] | 25.06 (4.14) 24.36 [19.12, 32.75] | 28.82 (8.11) 25.53 [18.04, 49.42] | 22.39 (2.61) 22.71 [18.76, 27.34] | 27.00 (6.88) 24.37 [16.78, 44.41] | 0.013 |
| Missing | 3 | 0 | 1 | 2 | 0 |  |
| *^1^* Mean (SD) Median [Min, Max]; n (%) | | | | | | |
| *^2^* Kruskal-Wallis rank sum test; Pearson’s Chi-squared test | | | | | | |

**Supplementary Table 2. EAA Regression Models in Table 3 Adjusted for Sociodemographic Covariates**

|  | | | | | | |
| --- | --- | --- | --- | --- | --- | --- |
|  | | | | | | |
|  | Dependent variable: | | | | | |
|  |  | | | | | |
|  | PC-Horvath 1 EAA | PC-Horvath 2 EAA | PC-Hannum EAA | PC-PhenoAge EAA | DunedinPACE (years) | PC-DNAm Telomere Length |
|  | (1) | (2) | (3) | (4) | (5) | (6) |
|  | | | | | | |
| HIV status: Participant with HIV (ref. Participant without HIV) | 4.703^***^ | 5.359^***^ | 3.636^***^ | 5.837^***^ | 0.046^*^ | -0.231^***^ |
|  | (0.868) | (0.952) | (0.893) | (1.117) | (0.022) | (0.031) |
|  |  |  |  |  |  |  |
| Ln-transformed High Sensitivity CRP | 0.043 | 0.101 | 0.075 | 0.464 | 0.014 | -0.006 |
|  | (0.281) | (0.308) | (0.289) | (0.361) | (0.007) | (0.010) |
|  |  |  |  |  |  |  |
| Chronological Age | -0.071 | -0.091 | -0.033 | -0.191 | 0.005 | 0.004 |
|  | (0.153) | (0.168) | (0.157) | (0.197) | (0.004) | (0.006) |
|  |  |  |  |  |  |  |
| Hispanic/Latinx (ref. African American/Black) | 2.004 | 2.874^*^ | 2.253 | 1.123 | 0.007 | -0.053 |
|  | (1.105) | (1.213) | (1.137) | (1.423) | (0.028) | (0.040) |
|  |  |  |  |  |  |  |
| Current Smoker: Yes (ref. No) | 1.001 | 1.438 | 0.524 | 1.895 | 0.039 | -0.014 |
|  | (0.876) | (0.962) | (0.902) | (1.128) | (0.022) | (0.032) |
|  |  |  |  |  |  |  |
| Marijuana Use: Yes (ref. No) | 0.275 | 0.521 | 0.087 | -0.072 | 0.003 | -0.025 |
|  | (1.135) | (1.245) | (1.168) | (1.460) | (0.028) | (0.041) |
|  |  |  |  |  |  |  |
| Substance Use: Yes (ref. No) | -1.980^*^ | -2.324^*^ | -2.059^*^ | -2.811^*^ | -0.006 | 0.038 |
|  | (0.890) | (0.977) | (0.916) | (1.146) | (0.022) | (0.032) |
|  |  |  |  |  |  |  |
| AUDIT Score | 0.024 | 0.046 | 0.027 | 0.028 | -0.001 | -0.0002 |
|  | (0.075) | (0.083) | (0.078) | (0.097) | (0.002) | (0.003) |
|  |  |  |  |  |  |  |
| PROMIS Depression T Score | 0.022 | 0.016 | 0.008 | -0.020 | -0.0005 | 0.001 |
|  | (0.054) | (0.060) | (0.056) | (0.070) | (0.001) | (0.002) |
|  |  |  |  |  |  |  |
| PSS Sum Score | -0.035 | -0.033 | 0.036 | 0.099 | 0.002 | -0.0004 |
|  | (0.086) | (0.095) | (0.089) | (0.111) | (0.002) | (0.003) |
|  |  |  |  |  |  |  |
| BMI | -0.034 | -0.057 | -0.064 | 0.031 | 0.003 | 0.002 |
|  | (0.075) | (0.082) | (0.077) | (0.096) | (0.002) | (0.003) |
|  |  |  |  |  |  |  |
| Constant | -0.860 | -0.473 | -0.710 | -0.322 | 0.796^***^ | -0.033 |
|  | (4.583) | (5.029) | (4.716) | (5.899) | (0.115) | (0.166) |
|  |  |  |  |  |  |  |
|  | | | | | | |
| Observations | 97 | 97 | 97 | 97 | 97 | 97 |
| Log Likelihood | -266.942 | -275.951 | -269.702 | -291.416 | 90.506 | 55.209 |
| Akaike Inf. Crit. | 557.883 | 575.902 | 563.405 | 606.833 | -157.012 | -86.418 |
|  | | | | | | |
| Note: | ^*^p<0.05; ^**^p<0.01; ^***^p<0.001 | | | | | |

**Supplementary Table 3. EAA Modeling in Table 3 Adjusted for Each of the 4 Principal Components**

|  | | | | | | |
| --- | --- | --- | --- | --- | --- | --- |
|  | | | | | | |
|  | Dependent variable: | | | | | |
|  |  | | | | | |
|  | PC-Horvath 1 EAA | PC-Horvath 2 EAA | PC-Hannum EAA | PC-PhenoAge EAA | PC-DNAm Telomere Length | PCDNAmTLResid |
|  | (1) | (2) | (3) | (4) | (5) | (6) |
|  | | | | | | |
| HIV status: Participant with HIV (ref. Participant without HIV) | 1.575 | 1.746 | 0.746 | 3.408^***^ | 0.036 | -0.068^**^ |
|  | (0.853) | (0.911) | (0.825) | (0.975) | (0.025) | (0.022) |
|  |  |  |  |  |  |  |
| Ln-transformed High Sensitivity CRP | 0.045 | -0.012 | -0.050 | 0.042 | 0.012^*^ | -0.003 |
|  | (0.199) | (0.212) | (0.192) | (0.227) | (0.006) | (0.005) |
|  |  |  |  |  |  |  |
| PC Score 1 | 1.652^***^ | 1.986^***^ | 1.652^***^ | 1.558^***^ | 0.004 | -0.084^***^ |
|  | (0.253) | (0.270) | (0.244) | (0.289) | (0.007) | (0.006) |
|  |  |  |  |  |  |  |
| PC Score 2 | -0.237 | -0.502 | -0.835^**^ | -1.938^***^ | -0.019^*^ | -0.003 |
|  | (0.285) | (0.304) | (0.276) | (0.326) | (0.008) | (0.007) |
|  |  |  |  |  |  |  |
| PC Score 3 | -0.125 | -0.115 | -0.891^*^ | -1.120^**^ | -0.001 | 0.012 |
|  | (0.353) | (0.376) | (0.341) | (0.403) | (0.010) | (0.009) |
|  |  |  |  |  |  |  |
| PC Score 4 | -0.138 | -0.095 | 0.219 | -0.006 | 0.021 | -0.026^*^ |
|  | (0.396) | (0.422) | (0.383) | (0.452) | (0.011) | (0.010) |
|  |  |  |  |  |  |  |
| Constant | -0.820 | -0.864 | -0.337 | -1.734^**^ | 1.008^***^ | 0.036^*^ |
|  | (0.570) | (0.609) | (0.551) | (0.652) | (0.016) | (0.014) |
|  |  |  |  |  |  |  |
|  | | | | | | |
| Observations | 100 | 100 | 100 | 100 | 100 | 100 |
| Log Likelihood | -262.208 | -268.741 | -258.846 | -275.547 | 92.472 | 105.693 |
| Akaike Inf. Crit. | 538.416 | 551.483 | 531.691 | 565.093 | -170.945 | -197.386 |
|  | | | | | | |
| Note: | ^*^p<0.05; ^**^p<0.01; ^***^p<0.001 | | | | | |

**Supplementary Table 4. EAA Regression Models in Supplementary Table 3 Adjusted for Sociodemographic Covariates**

|  | | | | | | |
| --- | --- | --- | --- | --- | --- | --- |
|  | Dependent variable: | | | | | |
|  |  | | | | | |
|  | PC-Horvath 1 EAA | PC-Horvath 2 EAA | PC-Hannum EAA | PC-PhenoAge EAA | DunedinPACE (years) | PC-DNAm Telomere Length |
|  | (1) | (2) | (3) | (4) | (5) | (6) |
|  | | | | | | |
| HIV status: Participant with HIV (ref. Participant without HIV) | 1.812 | 1.826 | 0.997 | 3.580^**^ | 0.038 | -0.065^**^ |
|  | (0.922) | (0.958) | (0.914) | (1.068) | (0.026) | (0.023) |
|  |  |  |  |  |  |  |
| Ln-transformed High Sensitivity CRP | -0.020 | -0.010 | -0.098 | 0.041 | 0.009 | -0.004 |
|  | (0.247) | (0.257) | (0.245) | (0.286) | (0.007) | (0.006) |
|  |  |  |  |  |  |  |
| Chronological Age | -0.109 | -0.129 | -0.074 | -0.187 | 0.005 | 0.006 |
|  | (0.132) | (0.137) | (0.131) | (0.153) | (0.004) | (0.003) |
|  |  |  |  |  |  |  |
| Hispanic/Latinx (ref. African American/Black) | 1.621 | 2.253^*^ | 1.394 | -0.623 | -0.014 | -0.029 |
|  | (0.971) | (1.009) | (0.963) | (1.125) | (0.027) | (0.024) |
|  |  |  |  |  |  |  |
| Current Smoker: Yes (ref. No) | 0.843 | 1.192 | 0.363 | 1.287 | 0.034 | -0.018 |
|  | (0.769) | (0.799) | (0.762) | (0.891) | (0.022) | (0.019) |
|  |  |  |  |  |  |  |
| Marijuana Use: Yes (ref. No) | -0.034 | 0.143 | 0.059 | 0.167 | 0.002 | -0.005 |
|  | (0.981) | (1.019) | (0.972) | (1.136) | (0.028) | (0.024) |
|  |  |  |  |  |  |  |
| Substance Use: Yes (ref. No) | -1.184 | -1.248 | -0.960 | -0.817 | 0.007 | 0.010 |
|  | (0.806) | (0.837) | (0.799) | (0.933) | (0.023) | (0.020) |
|  |  |  |  |  |  |  |
| AUDIT Score | -0.021 | -0.005 | -0.008 | -0.010 | -0.001 | 0.001 |
|  | (0.066) | (0.069) | (0.065) | (0.076) | (0.002) | (0.002) |
|  |  |  |  |  |  |  |
| PROMIS Depression T Score | 0.057 | 0.057 | 0.037 | 0.016 | -0.001 | 0.0001 |
|  | (0.048) | (0.050) | (0.047) | (0.055) | (0.001) | (0.001) |
|  |  |  |  |  |  |  |
| PSS Sum Score | -0.082 | -0.095 | -0.035 | -0.005 | 0.001 | 0.002 |
|  | (0.075) | (0.077) | (0.074) | (0.086) | (0.002) | (0.002) |
|  |  |  |  |  |  |  |
| BMI | -0.021 | -0.032 | -0.026 | 0.113 | 0.004^*^ | 0.001 |
|  | (0.065) | (0.067) | (0.064) | (0.075) | (0.002) | (0.002) |
| PC Score 1 | 1.558^***^ | 1.914^***^ | 1.542^***^ | 1.562^***^ | 0.005 | -0.084^***^ |
|  | (0.269) | (0.280) | (0.267) | (0.312) | (0.008) | (0.007) |
|  |  |  |  |  |  |  |
| PC Score 2 | 0.022 | -0.169 | -0.620 | -1.970^***^ | -0.024^**^ | -0.008 |
|  | (0.317) | (0.330) | (0.314) | (0.367) | (0.009) | (0.008) |
|  |  |  |  |  |  |  |
| PC Score 3 | -0.171 | -0.182 | -0.887^*^ | -1.213^**^ | 0.002 | 0.015 |
|  | (0.367) | (0.381) | (0.363) | (0.425) | (0.010) | (0.009) |
|  |  |  |  |  |  |  |
| PC Score 4 | -0.367 | -0.315 | -0.004 | 0.010 | 0.024 | -0.022^*^ |
|  | (0.436) | (0.452) | (0.432) | (0.505) | (0.012) | (0.011) |
|  |  |  |  |  |  |  |
|  |  |  |  |  |  |  |
| Constant | 0.562 | 1.021 | 0.556 | -1.110 | 0.777^***^ | -0.165 |
|  | (3.997) | (4.152) | (3.961) | (4.630) | (0.112) | (0.099) |
|  |  |  |  |  |  |  |
|  | | | | | | |
| Observations | 97 | 97 | 97 | 97 | 97 | 97 |
| Log Likelihood | -249.203 | -252.908 | -248.338 | -263.474 | 97.215 | 109.160 |
| Akaike Inf. Crit. | 530.407 | 537.815 | 528.675 | 558.947 | -162.430 | -186.319 |
|  | | | | | | |
| Note: | ^*^p<0.05; ^**^p<0.01; ^***^p<0.001 | | | | | |

**Supplementary Table 5. EAA Models Adjusted for T Lymphocytes**

|  | | | | | | |
| --- | --- | --- | --- | --- | --- | --- |
|  | Dependent variable: | | | | | |
|  |  | | | | | |
|  | PC-Horvath 1 EAA | PC-Horvath 2 EAA | PC-Hannum EAA | PC-PhenoAge EAA | DunedinPACE (years) | PC-DNAm Telomere Length |
|  | (1) | (2) | (3) | (4) | (5) | (6) |
|  | | | | | | |
| HIV status: Participant with HIV (ref. Participant without HIV) | 1.245 | 1.506 | 1.071 | 4.146^***^ | 0.047 | -0.045^*^ |
|  | (0.951) | (1.025) | (0.945) | (1.147) | (0.027) | (0.022) |
|  |  |  |  |  |  |  |
| Ln-transformed High Sensitivity CRP | 0.031 | -0.006 | -0.024 | 0.213 | 0.015^*^ | -0.002 |
|  | (0.202) | (0.218) | (0.201) | (0.244) | (0.006) | (0.005) |
|  |  |  |  |  |  |  |
| Memory CD4 T-cells (Salas) | -20.148 | -23.722 | -27.282^*^ | -29.569^*^ | 0.457 | 0.412 |
|  | (11.783) | (12.700) | (11.713) | (14.218) | (0.333) | (0.269) |
|  |  |  |  |  |  |  |
| Naive CD4 T-cells (Salas) | -33.423 | -45.049^*^ | -29.031 | -37.017 | -1.024^*^ | 1.388^***^ |
|  | (17.475) | (18.835) | (17.372) | (21.087) | (0.494) | (0.398) |
|  |  |  |  |  |  |  |
| Memory CD8 T-cells (Salas) | 15.680^**^ | 15.773^**^ | 5.538 | -6.866 | -0.222 | -1.071^***^ |
|  | (5.074) | (5.469) | (5.044) | (6.123) | (0.143) | (0.116) |
|  |  |  |  |  |  |  |
| Naive CD8 T-cells (Salas) | -18.191 | -29.861 | -57.675^**^ | -91.720^***^ | -0.746 | 1.445^**^ |
|  | (19.267) | (20.767) | (19.153) | (23.249) | (0.545) | (0.439) |
|  |  |  |  |  |  |  |
| Constant | 0.663 | 1.426 | 3.338^*^ | 4.197^**^ | 1.026^***^ | 0.044 |
|  | (1.315) | (1.417) | (1.307) | (1.587) | (0.037) | (0.030) |
|  |  |  |  |  |  |  |
|  | | | | | | |
| Observations | 100 | 100 | 100 | 100 | 100 | 100 |
| Log Likelihood | -264.588 | -272.087 | -263.998 | -283.378 | 92.021 | 113.501 |
| Akaike Inf. Crit. | 543.177 | 558.174 | 541.996 | 580.757 | -170.042 | -213.003 |
|  | | | | | | |
| Note: | ^*^p<0.05; ^**^p<0.01; ^***^p<0.001 | | | | | |

**Supplementary Figure 1. Correlation Plot of Leukocyte Composition Estimate Contribution to the Principal Components.** Note: Principal Components 1-4 explained 76.8% of variance in unadjusted model.
