# Supplementary figures and images for: Epigenetic Age Acceleration Is Associated With HIV Infection Independently of Inflammation
Source: Open Forum Infect Dis. 2026 May 5;13(5):ofag270. doi: 10.1093/ofid/ofag270 (PMC13195299; doi:10.1093/ofid/ofag270)

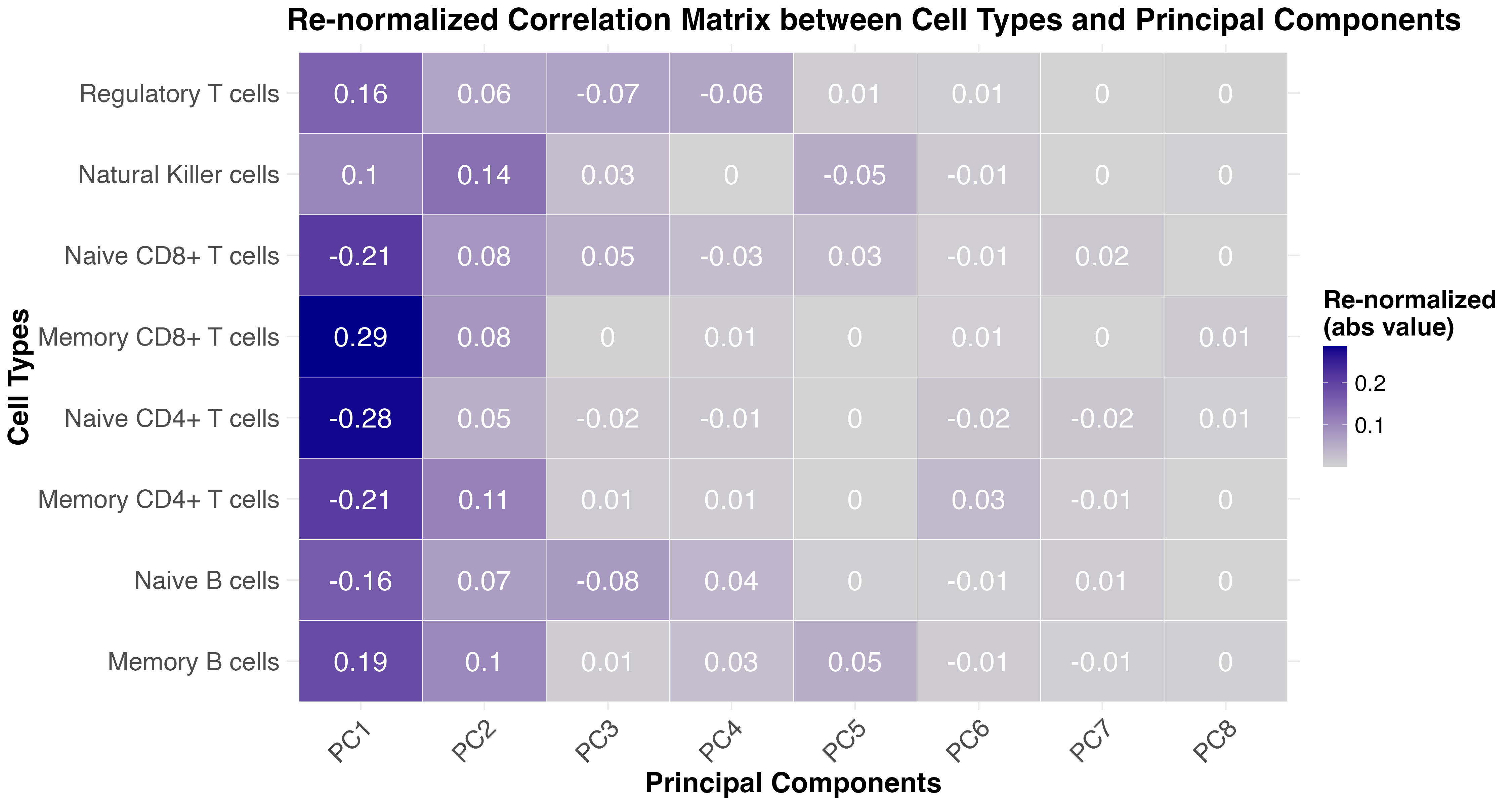

Supplement: ofag270_Supplementary_Data [file ofag270_supplementary_data.zip › Suupl Figure PCA_cells.heatmap.revised.jpeg]
